# Molecular profile of driver genes in lung adenocarcinomas of Brazilian patients who have never smoked: implications for targeted therapies

**DOI:** 10.1093/oncolo/oyae129

**Published:** 2024-06-29

**Authors:** Rodrigo de Oliveira Cavagna, Flávia Escremim de Paula, Gustavo Noriz Berardinelli, Murilo Bonatelli, Iara Santana, Eduardo Caetano Albino da Silva, Gustavo Ramos Teixeira, Beatriz Garbe Zaniolo, Josiane Mourão Dias, Flávio Augusto Ferreira da Silva, Carlos Eduardo Baston Silva, Marcela Gondim Borges Guimarães, Camila Pinto Barone, Alexandre Arthur Jacinto, Rachid Eduardo Noleto da Nóbrega Oliveira, José Elias Miziara, Pedro De Marchi, Miguel A Molina-Vila, Letícia Ferro Leal, Rui Manuel Reis

**Affiliations:** Molecular Oncology Research Center, Barretos Cancer Hospital, Barretos, Brazil; Molecular Diagnostic Laboratory, Barretos Cancer Hospital, Barretos, Brazil; Molecular Diagnostic Laboratory, Barretos Cancer Hospital, Barretos, Brazil; Molecular Diagnostic Laboratory, Barretos Cancer Hospital, Barretos, Brazil; Department of Pathology, Barretos Cancer Hospital, Barretos, Brazil; Department of Pathology, Barretos Cancer Hospital, Barretos, Brazil; Department of Pathology, Barretos Cancer Hospital, Barretos, Brazil; Barretos School of Health Sciences, Dr. Paulo Prata – FACISB, Barretos, Brazil; Molecular Oncology Research Center, Barretos Cancer Hospital, Barretos, Brazil; Barretos School of Health Sciences, Dr. Paulo Prata – FACISB, Barretos, Brazil; Department of Medical Oncology, Barretos Cancer Hospital, Barretos, Brazil; Department of Medical Oncology, Barretos Cancer Hospital, Barretos, Brazil; Department of Medical Oncology, Barretos Cancer Hospital, Barretos, Brazil; Department of Medical Oncology, Barretos Cancer Hospital, Barretos, Brazil; Department of Medical Oncology, Barretos Cancer Hospital, Barretos, Brazil; Deparment of Radiation Therapy, Barretos Cancer Hospital, Barretos, Brazil; Deparment of Thoracic Surgery, Barretos Cancer Hospital, Barretos, Brazil; Deparment of Thoracic Surgery, Barretos Cancer Hospital, Barretos, Brazil; Molecular Oncology Research Center, Barretos Cancer Hospital, Barretos, Brazil; Oncoclinicas, Rio de Janeiro, Brazil; Laboratory of Oncology/Pangaea Oncology, Dexeus University Hospital, Barcelona, Spain; Molecular Oncology Research Center, Barretos Cancer Hospital, Barretos, Brazil; Barretos School of Health Sciences, Dr. Paulo Prata – FACISB, Barretos, Brazil; Molecular Oncology Research Center, Barretos Cancer Hospital, Barretos, Brazil; Molecular Diagnostic Laboratory, Barretos Cancer Hospital, Barretos, Brazil; Life and Health Sciences Research Institute (ICVS), School of Medicine, University of Minho, Braga, Portugal; ICVS/3B’s—PT Government Associate Laboratory, Braga/Guimarães, Portugal

**Keywords:** lung adenocarcinoma, molecular profile, driver mutations, never smoker, Latin America

## Abstract

**Introduction:**

Lung cancer in never-smoker (LCINS) patients accounts for 20% of lung cancer cases, and its biology remains poorly understood, particularly in genetically admixed populations. We elucidated the molecular profile of driver genes in Brazilian LCINS.

**Methods:**

The mutational and gene fusion status of 119 lung adenocarcinomas from self-reported never-smoker patients, was assessed using targeted sequencing (NGS), nCounter, and immunohistochemistry. A panel of 46 ancestry-informative markers determined patients’ genetic ancestry.

**Results:**

The most frequently mutated gene was *EGFR* (49.6%), followed by *TP53* (39.5%), *ALK* (12.6%), *ERBB2* (7.6%), *KRAS* (5.9%), *PIK3CA* (1.7%), and less than 1% alterations in *RET*, *NTRK1*, *MET*∆ex14, *PDGFRA*, and *BRAF*. Except for *TP53* and *PIK3CA*, all other alterations were mutually exclusive. Genetic ancestry analysis revealed a predominance of European (71.1%), and a higher African ancestry was associated with *TP53* mutations.

**Conclusion:**

Brazilian LCINS exhibited a similar molecular profile to other populations, except the increased *ALK* and *TP53* alterations. Importantly, 73% of these patients have actionable alterations that are suitable for targeted treatments.

## Teaser

Lung cancer in never-smoker patients accounts for 20% of lung cancer cases, and its biology is poorly understood. This study focused on the molecular profile of driver genes in Brazilian patients who never smoked.

## Graphical Abstract

**Graphical Abstract:**
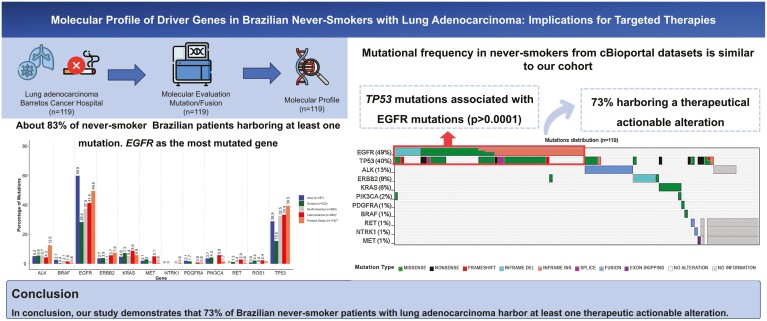

**Implications for Practice**

The identification of mutations in lung adenocarcinomas is crucial for deciding the best clinical management for the patients. Here, we observed 73% with at least one actionable alteration, with EGFR mutations reaching approximately 50% of patients. Therefore, these patients could be benefited by treatments with targeted drugs. A better understanding of the molecular profile in never-smoker patients from Brazil may improve the management of patients.

## Introduction

Lung cancer in patients who have never smoked (LCINS) accounts for 20% of lung cancer cases and remains underexplored despite its increasing worldwide incidence.^1-4^ Lung cancer in never-smokers shows a better prognosis compared to ever-smokers.^1-5^

Lung cancer biology varies between never-smokers and smokers.^1,3,6,7^ Lung adenocarcinomas in never-smoker patients exhibit a higher frequency of *EGFR, PIK3CA,* and *ERBB2* mutations.^1,6^*EGFR* mutations are notably more common, at variance with *KRAS* mutations, which are associated with tobacco exposure.^2-4^ Moreover, LCINS are more likely to harbor actionable variants, including not only *EGFR* mutations but also *ALK* translocation, impacting patients’ clinical management.^1,3^

Patient ethnicity also influences molecular profiles, with *EGFR* mutations more prevalent in Asians and *KRAS* mutations in Europeans.^3,8,9^ In admixed populations like Brazil, these profiles vary, and are poorly investigated.^10^ Therefore, we aimed to elucidate the molecular features of Brazilian LCINS.

## Materials and methods

### Study population

A series of 119 self-declared, never-smoker patients with lung adenocarcinoma (97 primary and 22 following treatment) from Barretos Cancer Hospital (BCH, Barretos, SP, Brazil) was evaluated. The local IRB approved the study (CAAE 05744712.3.0000.5437).

### Mutation detection

Tumor mutational analysis was performed in FFPE sections using the commercial panel TruSight Tumor 15 (Illumina, San Diego, CA, USA) on a MiSeq instrument. The read alignment and variant calling were performed with Sophia DDM software version 4.2 (Sophia Genetics SA, Lausanne, Switzerland). Variants were filtered out as previously described, and pathogenic variants were retained.^11,12^ Actionable mutations (Tier I and II) were determined as reported.^1^

### Fusion detection

*ALK* fusions were evaluated in 95.0% (*n* = 113/119) of cases by immunohistochemistry using the Ventana ALK (D5F3) CDx Assay (Roche, Basel, Switzerland) in an automated equipment.^11^

Detection of mRNA *ALK/RET/ROS1/NTRK1,2,3* fusion transcripts and *MET*∆ex14 (*MET* exon 14 skipping) was assessed in patients with *EGFR* and *KRAS* wild-type tumors (*n*=61) by the nCounter Elements XT (NanoString Technologies, Seattle, WA, USA) custom panel for *ALK*, *RET*, *ROS1*, and *NTRK1/2/3* fusion detection by specific partner and 3ʹ and 5ʹ disbalance, and *MET∆ex14,* using probes designed in-house.^13^ Twenty-four cases were inconclusive due to unavailable biological material. All analyses were performed in R environment v3.4.1.

### Genetic ancestry

The genetic ancestry background was evaluated using a set of 46 ancestry-informative markers, including INDELs for European (EUR), African (AFR), Asian (ASN), and Native American (AME).^8,12^

### Statistical analysis

We described categorical variables using percentages and continuous variables using the medians. Fisher’s exact test and χ^2^ test were conducted for the association between the *EGFR* and *TP53* mutations and genetic ancestry in IBM SPSS software Version 25 (IBM, Armonk, NY, USA) with a statistical significance limit of .05.

## Results

### Characterization of patients

The clinicopathological features of the 119 LCINS are summarized in Supplementary Table S1. Genetic ancestry was determined in 90.0% (*n* = 107/119) of the cases, following the proportion of 71.0% of EUR, 15.9% of AFR, 6.1% of ASN, and 7.9% of AME (Supplementary Figure S1).

### Molecular profile

**Figure 1.**
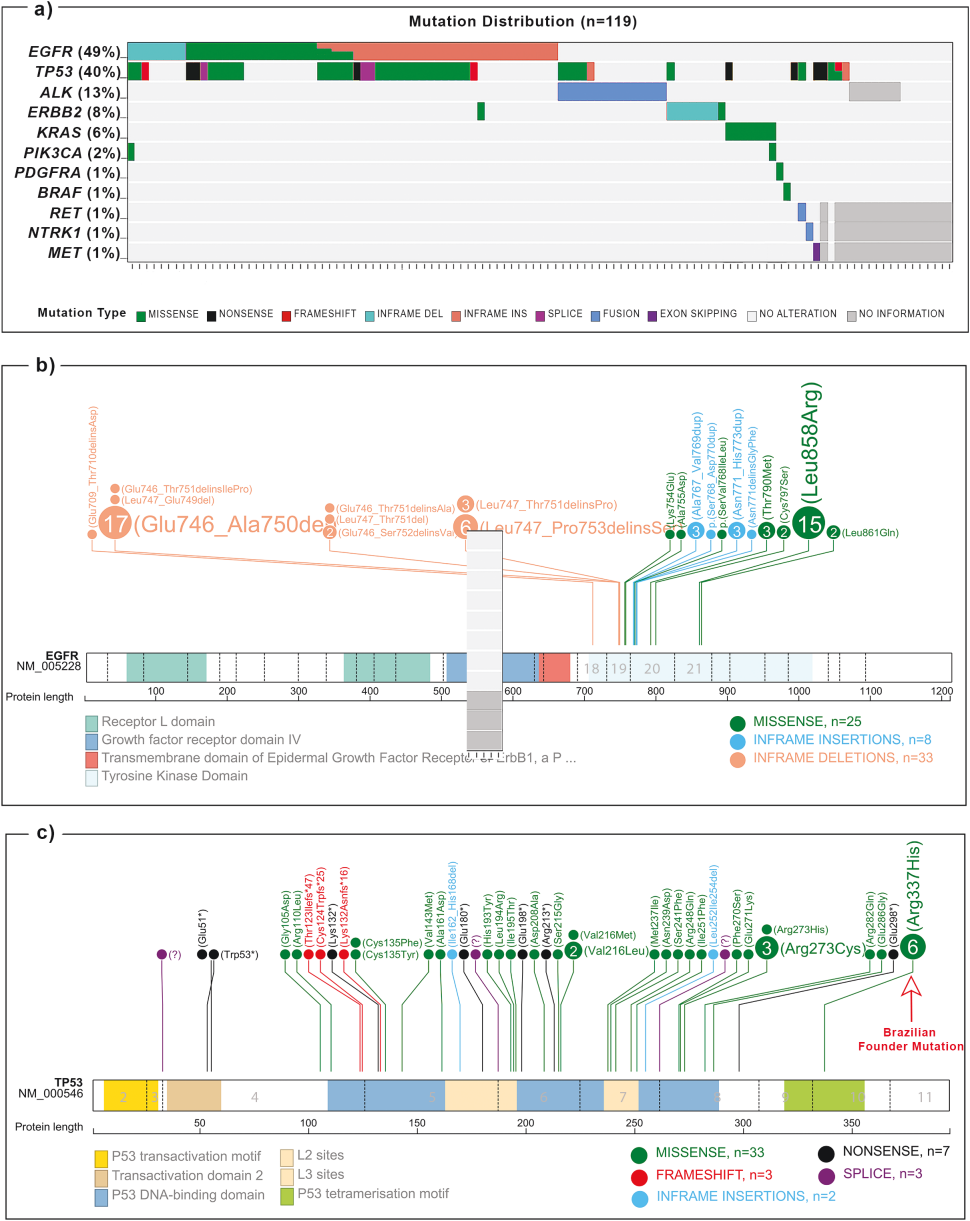
Representative figure of the mutations identified in the LCINS samples included in the study (*n* = 119). Only mutated genes are shown; (a) distribution of mutations through samples; lollipop plots depicting the distribution of (b) *EGFR* (*n* = 66); (c) *TP53* (*n* = 48). Figures were created using tools in https://proteinpaint.stjude.org/.

Among the 119 lung adenocarcinomas, 83.2% (*n* = 99/119) harbored at least one pathological mutation (Figure 1A). Among the 99 mutated cases, 54.5% carried one mutation, 39.4% 2, 4.1% 3, and 2.0% 4 (Supplementary Table S2). *EGFR* was the most mutated gene (49.6%, *n* = 59/119), followed by *TP53* 39.5% (*n* = 47/119), and *ALK* fusions in 12.6% (*n* = 15/119; Figure 1A). The genes *ERBB2*, *KRAS*, *PIK3CA*, *RET*, *BRAF*, *PDGFRA*, *NTRK1*, and *MET* showed fewer genetic alterations (Figure 1A). No alterations were observed on *AKT1*, *FOXL2*, *GNA11*, *GNAQ*, *KIT*, *NRAS*, *ROS1*, and *NTRK2/3* hotspot regions. Apart from *TP53* and *PIK3CA,* all the alterations were mutually exclusive (Figure 1A). Eighty-seven patients (73.1%) harbor actionable mutations (Supplementary Table S2)

Among *EGFR* mutations, exon 19 deletions were present in 49.2% of cases, followed by exon 21 (28.8%), and less frequently in exon 20 (7.6%) and 18 (1.7%; Figure 1B; Supplementary Table S2). Three tumors (following treatment) harbored the p.(Thr790Met) resistance mutation, and 2 presented additionally the p.(Cys797Ser) variant. Concerning *TP53*, the most common variant was the p.(Arg337His) (12.8%), followed by the p.(Arg273Cys) (6.4%) and the p.(Val216Leu) (4.3%; Figure 1C; Supplementary Table S2). One patient harbored 2 *TP53* variants (Supplementary Table S2). *TP53* mutations were associated with African ancestry (*P* = .002; Supplementary Table S3).

*E*xon 20 insertion p.(Tyr772_Ala775dup) accounted for 66.7% of *ERBB2* mutations (Supplementary Table S2). The most common variant of the *KRAS* gene was p.(Gly12Asp) (42,8%, *n* = 3/7), and the variants observed in the *PIK3CA*, *BRAF*, and *PDGFRA* genes were at hotspot regions (Supplementary Table S2).

ALK fusions were observed in 15 patients (12.6%), both by immunohistochemistry and nCounter, the latter allowing to identify *EML4* as the fusion partner in 73.3% of the cases. *RET* and *NTRK1* fusions were identified by 3ʹ-5ʹ disbalance in one patient each, (Figure 1A; Supplementary Table S2).

### Co-occurring mutations

*EGFR* and *TP53* mutations significantly co-occurred in 27.7% (*n* = 33/119) of cases (*P* < .0001; Figure 1A; Supplementary Table S4). The Brazilian founder *TP53* mutation p.(Arg337His) variant was mostly concurrent with *EGFR* mutations (Supplementary Table S2). *EGFR* variants also co-occurred with *PIK3CA* and *ERBB2* mutations*. TP53* mutations co-occurred with 1/3 of *ALK* fusions. The *PIK3CA* variants also co-occurred with the *KRAS* and *TP53* (Figure 1A).

## Discussion

The present study interrogated the molecular profile of driver genes of LCINS from a single Brazilian institution. Overall, we found that 73% of cases harbor actionable molecular alterations, in accordance with the literature.^1,4,5^

**Figure 2.**
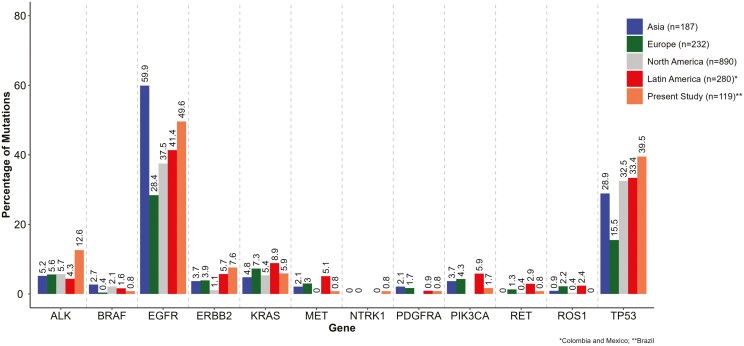
Mutational frequency comparison between studies with never-smoker patients with lung adenocarcinoma according to the geographic region of the study (*n* = 1708).

*EGFR* mutations occurred in half of our cases, in agreement with other populations, being higher than European and lower than Asian populations (Figure 2; Supplementary Table S5).^1,2,4,6^ Similar to our results, EGFR-TKi sensitizing exon 19 deletions are found in 50% of patients diagnosed with lung adenocarcinoma, while exon 20 insertions are less common globally.^1,6,8^*TP53* was our second most mutated gene (39.5%). This frequency is higher compared to studies of LCINS in Asia, Europe, North America, and Latin America (Figure 2; Supplementary Table S5).^6^ We observed an association between *TP53* mutations and higher African ancestry, similar to our recent study on ever- and never-smoker patients with lung adenocarcinoma.^12^ Notably, the most frequent *TP53* mutation was the Brazilian germline variant p.(Arg337His), often concurrent with *EGFR* mutations. Previous lung cancer studies reported higher co-occurrence of *EGFR* and *TP53* mutations, mainly with the Brazilian founder mutation.^14-17^

We observed 7.6% of *ERBB2* mutations in our LCINS, consistent with 2%-13% reported in other populations (Figure 2; Supplementary Table S5).^1-4,6^ In our series, only 5.9% of patients with lung adenocarcinoma had *KRAS* mutations, in accordance with other LCINS studies (4.4%-18%). Interestingly, *ALK* fusions in our LCINS series were significantly more frequent (12.6%) than reported globally (3%-8%).^2,4,6^ Finally, as previously reported, we identified less frequent alterations (1%-2%) in the genes *PIK3CA*, *PDGFRA*, *BRAF*, *RET*, *NTRK1*, and *MET,* similar to other populations^1,6,18^ (Figure 2; Supplementary Table S5).

Thus, we observe in the Brazilian LCINS population an overall similarity in the frequencies of driver genes reported worldwide. The exception was our higher frequency of *ALK* fusions and *TP53* mutations, which could potentially be due to the significant presence of African ancestry, or founder *TP53* p.(Arg337His) variant in the Brazilian population (Figure 2; Supplementary Table S5). Further studies are needed to validate and extend these findings.

Concluding, the molecular profile of Brazilian LCNIS resembles that of other populations worldwide, and 73% of patients could be eligible for personalized treatments.

## Supplementary material

Supplementary material is available at *The Oncologist* online.

oyae129_suppl_Supplementary_Figure_S1

oyae129_suppl_Supplementary_Table_1

oyae129_suppl_Supplementary_Table_2

oyae129_suppl_Supplementary_Table_3

oyae129_suppl_Supplementary_Table_4

oyae129_suppl_Supplementary_Table_5

## Data Availability

The data that support the findings of this study are available from Dr. Rui Manuel Reis, but restrictions apply to the availability of these data, because of patients’ personal data. De-identified data are, however, available from the authors upon request.
