# Supplementary material for: Molecular profile of driver genes in lung adenocarcinomas of Brazilian patients who have never smoked: implications for targeted therapies
Source: Oncologist. 2024 Jun 29;29(10):e1419–24. doi: 10.1093/oncolo/oyae129 (PMC11449088; doi:10.1093/oncolo/oyae129)
Supplement: oyae129_suppl_Supplementary_Figure_S1 [file oyae129_suppl_supplementary_figure_s1.docx]

**Supplementary Figure Caption**

**Supplementary Figure 1 -** Genetic ancestry proportions from lung adenocarcinomas (n=107). Figure shows the ancestry proportions in the X axis and the ancestry proportion of the populations for each patient in the Y axis. AFR, African (red); EUR, European (green); ASN, Asian (blue); AME, Native American (yellow).

**
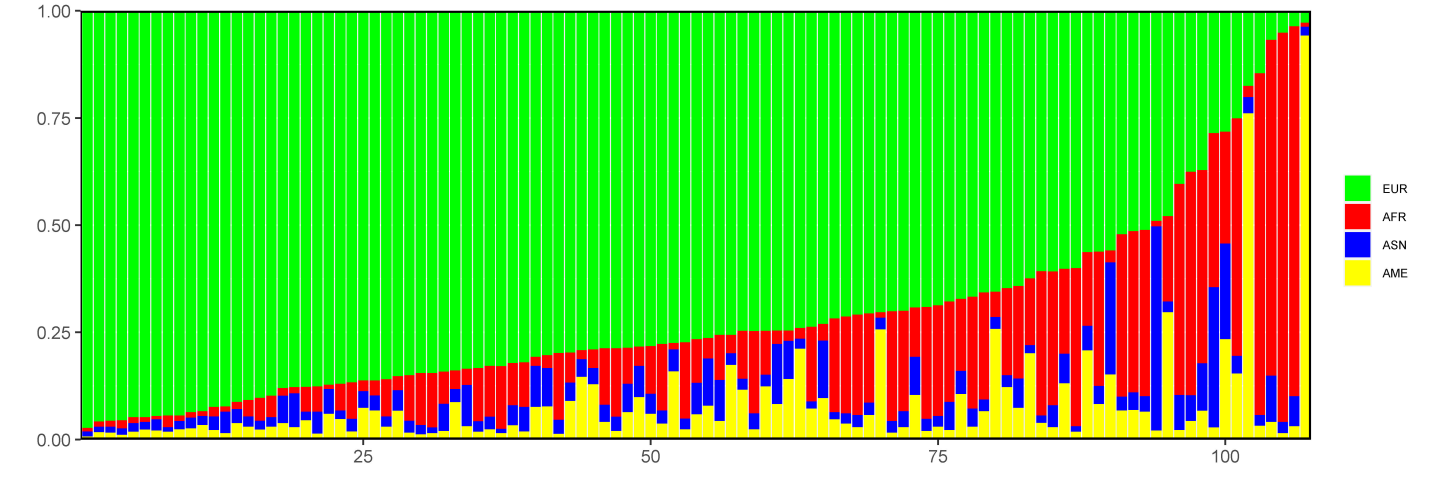
**
