# Supplementary material for: Molecular profile of driver genes in lung adenocarcinomas of Brazilian patients who have never smoked: implications for targeted therapies
Source: Oncologist. 2024 Jun 29;29(10):e1419–24. doi: 10.1093/oncolo/oyae129 (PMC11449088; doi:10.1093/oncolo/oyae129)
Supplement: oyae129_suppl_Supplementary_Table_1 [file oyae129_suppl_supplementary_table_1.docx]

|  |  | Adenocarcinoma (n=119) | |
| --- | --- | --- | --- |
|  |  |  |  |
| Characteristics | **Parameters** | **n** | **(%)** |
| Age (year) | Median (range) | 60 | (19 – 86) |
|  | <=60 | 62 | (52.1) |
|  | >60 | 57 | (47.9) |
| Sex | Female | 77 | (64.7) |
|  | Male | 42 | (35.3) |
| Self-reported skin color* | White | 69 | (58.0) |
|  | Non-White | 26 | (21.8) |
|  | Missing | 24 | (20.2) |
| ECOG PS | 0 | 27 | (22.7) |
|  | 1 | 51 | (42.9) |
|  | 2 | 23 | (19.3) |
|  | 3/4 | 7 | (5.9) |
|  | Missing | 11 | (9.2) |
| Weight loss** | No | 42 | (35.3) |
|  | <=10% of weight | 36 | (30.2) |
|  | >10% of weight | 22 | (18.5) |
|  | Missing | 19 | (16.0) |
| Stage at diagnosis*** | I/II/III | 29 | (24.4) |
|  | IV | 86 | (72.3) |
|  | Missing | 4 | (3.4) |
| Metastasis at diagnosis | No | 29 | (24.4) |
|  | Yes, CNS | 29 | (24.4) |
|  | Yes, Others | 56 | (47.1) |
|  | Missing | 5 | (4.2) |
| Vital status | Alive – no disease | 8 | (6.7) |
|  | Alive – active disease | 35 | (29.4) |
|  | Death - cancer | 72 | (60.5) |
|  | Death - others | 3 | (2.5) |
|  | Loss of follow-up | 1 | (0.8) |

**Supplementary Table 1** – Clinicopathological and molecular features of Brazilian never-smokers lung cancer patients

*According to IBGE (Instituto Brasileiro de Geografia e Estatística); ** Weight of loss 6 months prior to diagnosis. ***According to AJCC 7^th^ edition.
