# Supplementary material for: Molecular profile of driver genes in lung adenocarcinomas of Brazilian patients who have never smoked: implications for targeted therapies
Source: Oncologist. 2024 Jun 29;29(10):e1419–24. doi: 10.1093/oncolo/oyae129 (PMC11449088; doi:10.1093/oncolo/oyae129)
Supplement: oyae129_suppl_Supplementary_Table_3 [file oyae129_suppl_supplementary_table_3.docx]

**Supplementary Table 3 –** Association between most frequent molecular alterations and genetic ancestry background (n=119).

|  | | ***TP53* status** | | | | | | | ***EGFR* status** | | | | | | ***ALK* status*** | | | | | | | | ***ERBB2* status** | | | |  |
| --- | --- | --- | --- | --- | --- | --- | --- | --- | --- | --- | --- | --- | --- | --- | --- | --- | --- | --- | --- | --- | --- | --- | --- | --- | --- | --- | --- |
| **Ancestry Tertiles** | **Wild-type** | | | | **Mutated** | | |  | | **Wild-type** | | **Mutated** | |  | | | **Wild-type** | | **Mutated** | | |  | **Wild-type** | | **Mutated** | |  |
|  | **n** | | | **%** | **n** | | **%** | **p-value** | | **n** | **%** | **n** | **%** | **p-value** | | | **n** | **%** | **n** | | **%** | **p-value** | **n** | **%** | **n** | **%** | **p-value** |
| **African** | |  |  | | |  |  |  |  | |  |  |  |  | |  | |  |  |  | |  |  |  |  |  |  |
| **Low** | | 29 | 82.9 | | | 6 | 17.1 | **0.002** | 23 | | 65.7 | 12 | 34.3 | 0.063 | | 26 | | 83.9 | 5 | 16.1 | | 0.320 | 31 | 88.6 | 4 | 11.4 | 0.632 |
| **Intermedium** | | 19 | 52.8 | | | 17 | 47.2 |  | 16 | | 44.4 | 20 | 55.6 |  |  | 29 | | 82.9 | 6 | 17.1 | |  | 33 | 91.7 | 3 | 8.3 |  |
| **High** | | 16 | 44.4 | | | 20 | 55.6 |  | 14 | | 38.9 | 22 | 61.1 |  |  | 32 | | 94.1 | 2 | 5.9 | |  | 34 | 94.4 | 2 | 5.6 |  |
| **Missing** | | 8 |  | | | 4 |  |  | 7 | |  | 5 |  |  | | 9 | |  | 2 |  | |  | 12 |  | 0 |  |  |
| **Asian** | |  |  | | |  |  |  |  | |  |  |  |  | |  | |  |  |  | |  |  |  |  |  |  |
| **Low** | | 25 | 71.4 | | | 10 | 28.6 | 0.250 | 18 | | 51.4 | 17 | 48.6 | 0.912 | | 30 | | 93.8 | 2 | 6.3 | | 0.421 | 31 | 88.6 | 4 | 11.4 | 0.629 |
| **Intermedium** | | 20 | 54.1 | | | 17 | 45.9 |  | 17 | | 45.9 | 20 | 54.1 |  |  | 29 | | 85.3 | 5 | 14.7 | |  | 35 | 94.6 | 2 | 5.4 |  |
| **High** | | 19 | 54.3 | | | 16 | 45.7 |  | 18 | | 51.4 | 17 | 48.6 |  |  | 28 | | 82.4 | 6 | 17.6 | |  | 32 | 91.4 | 3 | 8.6 |  |
| **Missing** | | 8 |  | | | 4 |  |  | 7 | |  | 5 |  |  | | 9 | |  | 2 |  | |  | 12 |  | 0 |  |  |
| **European** | |  |  | | |  |  |  |  | |  |  |  |  | |  | |  |  |  | |  |  |  |  |  |  |
| **Low** | | 17 | 48.6 | | | 18 | 51.4 | 0.139 | 17 | | 48.6 | 18 | 51.5 | 1 | | 32 | | 94.1 | 2 | 5.9 | | 0.096 | 31 | 88.6 | 4 | 11.4 | 0.177 |
| **Intermedium** | | 25 | 59.5 | | | 17 | 40.5 |  | 21 | | 50.0 | 21 | 50.0 |  |  | 31 | | 77.5 | 9 | 22.5 | |  | 41 | 97.6 | 1 | 2.4 |  |
| **High** | | 22 | 73.3 | | | 8 | 26.7 |  | 15 | | 50.0 | 15 | 50.0 |  |  | 24 | | 92.3 | 2 | 7.7 | |  | 26 | 86.7 | 4 | 13.3 |  |
| **Missing** | | 8 |  | | | 4 |  |  | 7 | |  | 5 |  |  | | 9 | |  | 2 |  | |  | 12 |  | 0 |  |  |
| **Native American** | | | | | | | |  |  | |  |  |  |  | |  | |  |  |  | |  |  |  |  |  |  |
| **Low** | | 22 | 56.4 | | | 17 | 43.6 | 0.562 | 21 | | 53.8 | 18 | 46.2 | 0.831 | | 30 | | 83.3 | 6 | 16.7 | | 0.807 | 36 | 92.3 | 3 | 7.7 | 1 |
| **Intermedium** | | 16 | 55.2 | | | 13 | 44.8 |  | 14 | | 48.3 | 15 | 51.7 |  |  | 25 | | 89.3 | 3 | 10.7 | |  | 26 | 89.7 | 3 | 10.3 |  |
| **High** | | 26 | 66.7 | | | 13 | 33.3 |  | 18 | | 46.2 | 21 | 53.8 |  |  | 32 | | 88.9 | 4 | 11.1 | |  | 36 | 92.3 | 3 | 7.7 |  |
| **Missing** | | 8 |  | | | 4 |  |  | 7 | |  | 5 |  |  | | 9 | |  | 2 |  | |  | 12 |  | 0 |  |  |

**ALK* status (n=111). Inconclusive samples were not considered here. P-value = χ^2^ test
