# Supplementary material for: Molecular profile of driver genes in lung adenocarcinomas of Brazilian patients who have never smoked: implications for targeted therapies
Source: Oncologist. 2024 Jun 29;29(10):e1419–24. doi: 10.1093/oncolo/oyae129 (PMC11449088; doi:10.1093/oncolo/oyae129)
Supplement: oyae129_suppl_Supplementary_Table_4 [file oyae129_suppl_supplementary_table_4.docx]

|  | ***TP53* status** | | | | |
| --- | --- | --- | --- | --- | --- |
| ***EGFR* status** | **Wild-type** | | **Mutated** | |  |
|  | **n** | **%** | **n** | **%** | **p-value** |
| **Wild-type** | 46 | 76.7 | 14 | 23.3 | **<0.0001** |
| **Mutated** | 26 | 44.1 | 33 | 55.9 |  |

**Supplementary Table 4 –** Association between *EGFR* mutations and *TP53* mutations (n=119).
